# Enhancing On-Call Preparedness Among Junior Doctors in Psychiatry: A Comprehensive Bootcamp Approach

**DOI:** 10.1192/bjo.2024.324

**Published:** 2024-08-01

**Authors:** Ananya Santosh, Praveen Kumar, Susan Brown

**Affiliations:** New Craig's Psychiatric Hospital, Inverness, United Kingdom

## Abstract

**Aims:**

The Psychiatry Bootcamp at a psychiatric hospital was developed to address the unique and diverse challenges faced by new trainees, including Foundation Year Doctors, General Practitioner Specialty Trainees, Core Psychiatry trainees and Broad-Based Trainees, during their rotations in psychiatry in the Highlands, Scotland. The aim was to enhance their core skills and confidence levels, ensuring they are well-prepared for their first on-call shift. This initiative seeks to complement the existing induction program, specifically targeting areas of acute medical and psychiatric emergencies and care, that are critical for on-call duties.

**Methods:**

Since its launch in 2022, the Psychiatry Bootcamp has been conducted quarterly, aligning with new doctor rotations. Held at the Medical Education Centre/Psychiatry Hospital, this one-day intensive training accommodates an average of 10 participants per session. The program, delivered by consultants, specialist nurses, and senior trainees, comprises tutorials, practical skills sessions, and simulated scenarios, focusing on key areas like the Mental Health Act, psychiatric risk assessment, wound management, resuscitation guidelines, and rapid tranquilisation.

A pre-test is administered to gauge participants' baseline knowledge and skills. Feedback is also collected immediately after the session and 3–4 months later. This ongoing feedback, systematically gathered since 2022, has been pivotal in continuously refining the curriculum and teaching methods, ensuring they remain up-to-date and effective.

**Results:**

The bootcamp demonstrated notable success in enhancing the preparedness of new psychiatry trainees for on-call duties. Post-course evaluations revealed an improvement in participants' confidence levels when managing psychiatric emergencies and various on-call situations. Through the practical and interactive nature of the training, trainees reported a deeper understanding of acute psychiatric care and an increased ability to apply theoretical knowledge in real-life scenarios. The hands-on experience with simulated scenarios was particularly effective in bridging the gap between classroom learning and clinical practice. Trainees expressed greater comfort in handling challenging situations, such as rapid tranquilisation and emergency detention under the Mental Health Act, which were previously areas of concern.

**Conclusion:**

The Psychiatry Bootcamp represents a targeted and effective approach to preparing new psychiatry trainees for the demands of on-call duties. By focusing on key areas of need and employing a variety of teaching methods tailored to enhance practical skills and confidence, the bootcamp successfully addresses the gap between theoretical knowledge and clinical application. Preliminary feedback underscores the value of such programs in psychiatric education, suggesting that this model could be beneficial for similar settings seeking to improve trainee preparedness and overall patient care quality.

